# Biobased polyester versus synthetic fiberglass casts for treating stable upper limb fractures in children: a randomized controlled trial

**DOI:** 10.1186/s12891-023-07138-7

**Published:** 2024-01-02

**Authors:** Tsung-Yu Lan, Chin-Wen Chen, Yu-Hao Huang, Shang-Ming Lin, Ching-Ting Liang, Chih-Hung Chang, Syang-Peng Rwei

**Affiliations:** 1 Department of Orthopedic Surgery, Far Eastern Memorial Hospital, No. 21, Sec. 2, Nanya S. Rd, New Taipei City, 220 Taiwan (R.O.C.); 2 Department of Materials and Textiles, Asia Eastern University of Science and Technology, No.58, Sec.2, Sihchuan Rd, New Taipei City, 220 Taiwan (R.O.C.); 3 Institute of Organic and Polymeric Materials, National Taipei University of Technology, 1, Sec. 3, Zhongxiao E. Rd, Taipei, 10608 Taiwan (R.O.C.); 4 Research and Development Center for Smart Textile Technology, National Taipei University of Technology, 1, Sec. 3, Zhongxiao E. Rd, Taipei, 10608 Taiwan (R.O.C.); 5 Graduate School of Biotechnology and Bioengineering, Yuan Ze University, Taoyuan, Taiwan (R.O.C.); 6 Department of Molecular Science and Engineering, Institute of Organic and Polymeric Materials, Research and Development Center of Smart Textile Technology, National Taipei University of Technology, No. 1, Sec. 3, Chung-Hsiao East Road, Taipei, 106 Taiwan (R.O.C.)

**Keywords:** Pediatric upper limb fracture, Synthetic fiberglass cast, Biobased polyester cast

## Abstract

**Background:**

Stable upper limb fractures, such as radius, ulna, or distal humerus fractures, are common pediatric orthopedic traumas that are traditionally managed with cast immobilization. The commonly used synthetic fiberglass cast is light and water resistant but may promote skin itchiness during casting, which is a common complaint of patients. In addition, these diisocyanate-based casts have been proven to be toxic and may cause asthma. Herein, we introduce a novel biobased polyester cast to compare its clinical outcomes and patient satisfaction with conventional synthetic fiberglass casts.

**Methods:**

From Feb 2022 to Nov 2022, we undertook a single-center prospective randomized trial involving 100 children with cast-immobilized stable upper limb fractures. These patients were randomized into either biobased polyester or synthetic fiberglass groups. All patients were regularly followed up till the cast removal which occurred approximately 3–4 weeks after immobilizing. Objective clinical findings and subjective patient questionnaire were all collected and analyzed.

**Results:**

According to the radiographs taken on the day of cast removal, there was no loss of reduction in both groups. The incidence of skin problems was 3.4 times higher in the synthetic fiberglass group than in the biobased polyester group. For the subjective questionnaire, the biobased polyester cast was preferred in every sub-item.

**Conclusions:**

Our study strongly suggested that the novel biobased polyester cast provides matching stability to conventional fiberglass casts and improves patient satisfaction in an eco-friendlier and safer way.

**Trial registration:**

ClinicalTrials.gov Protocol Registration and Results System (https://www.clinicaltrials.gov/; ID: NCT06102603; Date: 26/10/2023).

## Background

Stable upper limb fractures, such as radius, ulna, or distal humerus fractures, are common pediatric orthopedic traumas that are traditionally managed with cast immobilization [1–3]. The synthetic fiberglass cast composed of polyurethane and diisocyanate has been used worldwide and has superior patient satisfaction and clinical outcomes than plaster of Paris (POP) casts [4, 5]. Synthetic fiberglass casts are typically activated by water, resulting in a lightweight cast that offers enhanced strength and water resistance [6]. However, skin itchiness or irritation during the entire casting period is a common complaint of patients. In contrast, the materials in synthetic fiberglass casts have drawbacks: diisocyanate was proven to cause asthma [7] and polyurethane is not a biodegradable material and could increase the environmental burden. As the materials are not recyclable, the use of petroleum-based polymers has led to concerns worldwide [8]. In response, biobased materials have attracted considerable attention. A novel biobased cast design with larger ventilation holes [8] could theoretically decrease skin irritation, itchiness, and bad odors. This study aimed to evaluate patient satisfaction and clinical outcomes when casting with a novel biobased polymer versus synthetic fiberglass. We hypothesized that, compared to a synthetic fiberglass cast, immobilization using a biobased polyester cast could result in better patient satisfaction and achieve similar clinical outcomes in children with stable upper limb fractures.

## Methods

The clinical trial was approved by the research ethics review committee of the author’s hospital (Number 110,245-F) and informed consent was obtained from the legal guardian of each patient. The trial was registered at ClinicalTrials.gov Protocol Registration and Results System (https://www.clinicaltrials.gov/; ID: NCT06102603; Date: 27/01/2022). Between February 2022 and November 2022, all children (age range, 4–15 years) with radiography-diagnosed stable upper limb fractures (including radius, ulna, and distal humerus fractures such as Garland type I supracondylar fractures of the humerus) [9] diagnosed at the orthopedic outpatient clinic or emergency room of a tertiary trauma center hospital were eligible for recruitment. Exclusion criteria included displaced or unstable fractures indicated for close or open reduction and fixation, previous surgeries to the affected upper limb, history of any chronic skin pathology (e.g., atopic dermatitis), and known allergy to cast materials.

**Fig. 1:**
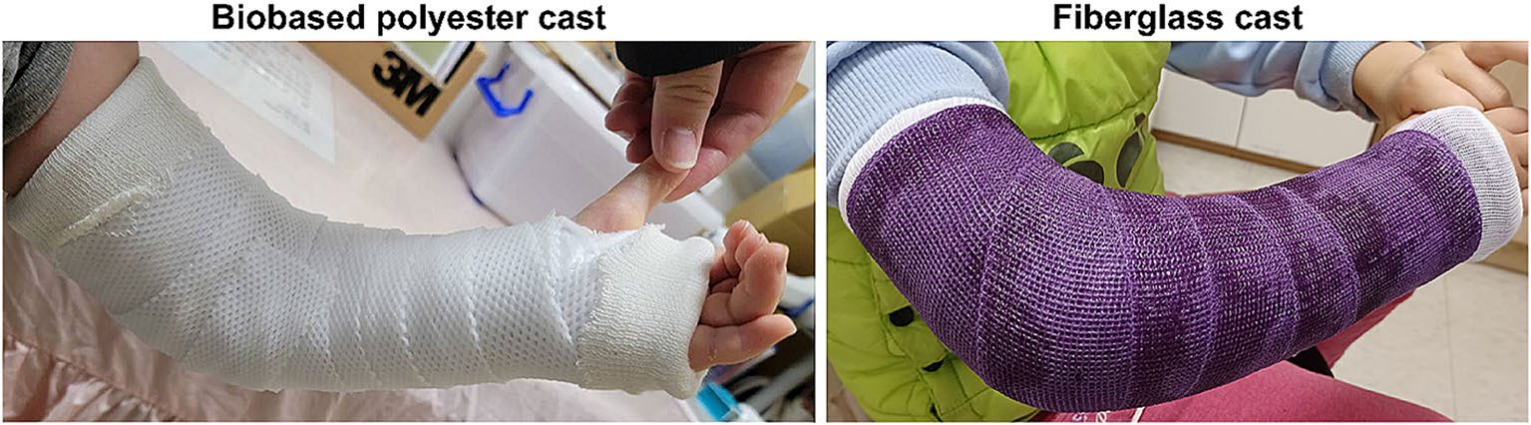
Images of biobased polyester and fiberglass casts being worn by patients

Patients were randomized using a sealed envelope to either the biobased polyester cast group (MEDlite Thermo Casting Tape, Taipei Smart Materials, Taipei, Taiwan) or the fiberglass cast group (Scotchcast, 3 M Health Care, Maplewood, Minnesota, U.S.) (Fig. 1). A well-trained technician applied the long arm cast to the patients. Before the cast was applied, the same type of cotton stockinet and cotton padding was applied to all patients. In the biobased group, the cast was immersed in a bowl of water heated to around 60 °C for 3 min. Once the cast bandage had become pliable and soft, it was then wrapped around the affected limb. Typically, the biobased cast can retain its plasticity until it cools down, allowing us approximately 5–7 min to finalize the casting process depending on the room temperature. The elbow was flexion up to 90° and in a neutral position. Both groups were instructed not to let the cast become wet and cast care guidelines were given to patients and parents. The cast was kept on for approximately 3–4 weeks.

Standard radiographs of the affected region were taken at 1-week post-injury to control secondary displacement. All patients were reviewed for cast removal after 3 to 4 weeks, and standard radiographs were taken again. One author reviewed the radiography images. Complications, such as loss of reduction and further need for close or open reduction, nonunion, malunion, cubitus varus neurovascular injury, and any skin complication, such as rash or growth of hairs, were documented. The patient satisfaction questionnaire was completed at the time of cast removal with or without the help of parents or guardians. The questionnaire (1 [lowest] to 5 [highest]) included questions to rate comfort, weight, itchiness, heat retention, smell, and total satisfaction. It was not age-dependent and was easily understood by children (Table 1) [4]. The score of the questionnaire was recorded by a study nurse who was not aware of which type of casting material was used.

**Table 1:** Patient satisfaction questionnaire

Question	Response				
	1	2	3	4	5
Over the last week, has your cast been comfortable?	Want it removed	Irritating	Fairly comfortable, Occasional irritation	Comfortable	Very comfortable
When your cast was first applied?	Uncomfortable	Took up to a week to become comfortable	Fairly comfortable after a few days	Took 1 to 2 days to become comfortable	Very easy to get used to
Weight of cast	Heavy cast. Difficult to use arm	Moderately heavy. Limited multiple activities	Mildly heavy. Limited several activities	Fairly light	Light cast. Did not interfere with activity
Itchiness	Very itchy	Frequent itch but tolerable	Sometimes itchy	Rarely itchy	No itch
Hot and sweaty	Very hot. Wanted cast removed	Hot feeling worrying. Complained a lot	Often hot. Mildly distressing	Hot at times, but tolerable	Well-tolerated
Smell	Distressing smell	Continual mild odor	Smelly after a hot day	Occasionally smelly	None
Overall satisfaction	Awful, intolerable	OK, not as easy as imagined	Good overall comfort	Very comfortable	Excellent, would recommend to friends

Statistical analyses were performed using the Mann–Whitney *U* test for continuous variables and Fisher’s exact test for categorial variables. A p-value < 0.05 was considered statistically significant.

## Results

**Fig. 2:**
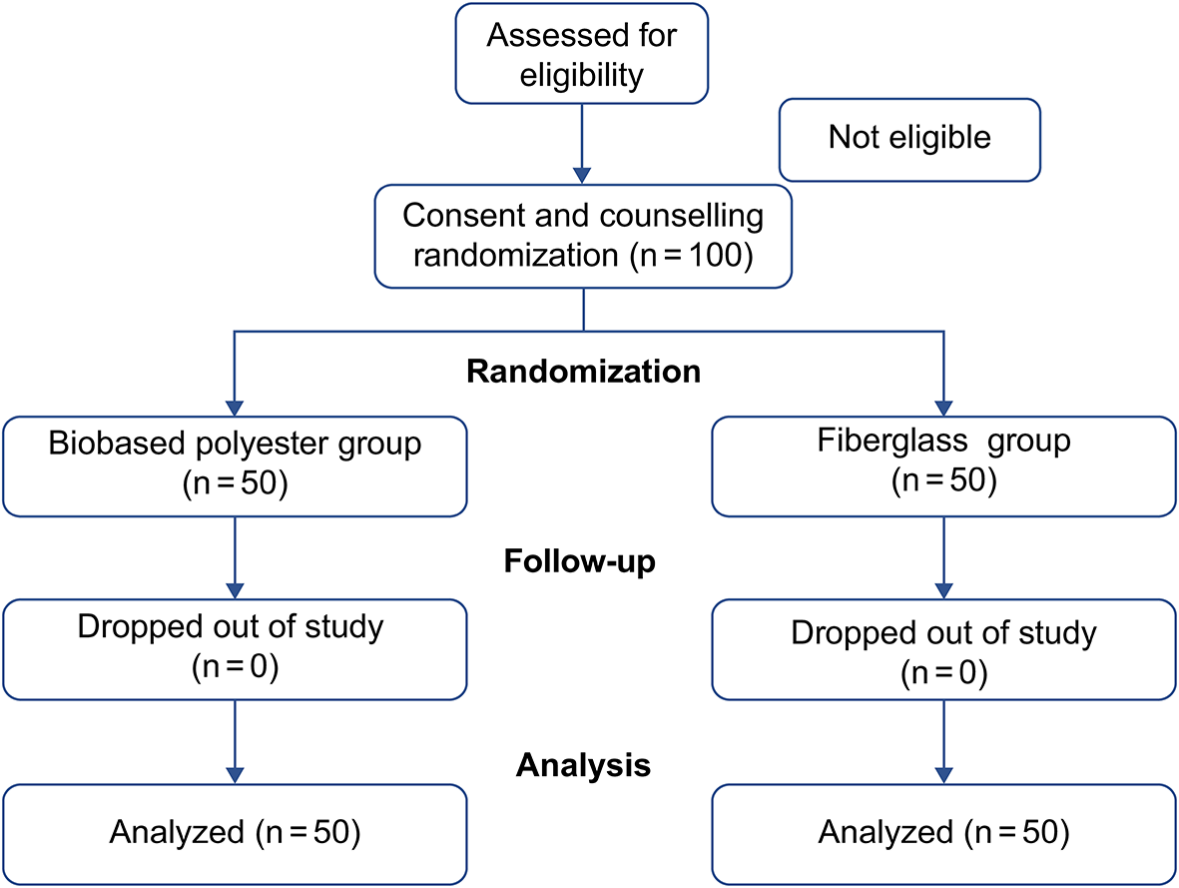
Flow diagram shows the progress of all patients through each stage

A total of 100 patients were included in this study. Patients were randomized into either the biobased polyester (n = 50) or fiberglass (n = 50) cast group. No patients dropped out of the study and all patients completed the questionnaire (Fig. 2).

**Fig. 3:**
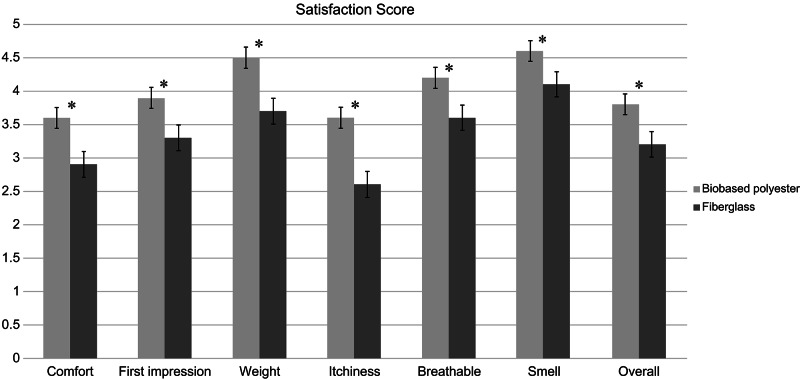
Bar chart of mean patient satisfaction score in biobased polyester cast and fiberglass cast groups. The error bars denote standard error of the mean. The asterisks denote statistical significance (p < 0.05)

The demographic data showed no statistical differences between the two groups for age, sex, fracture site distribution, or casting duration (Table 2). The mean patient satisfaction scores for each category and total satisfaction were significantly higher in the biobased polyester cast group than for the synthetic fiberglass cast group (Fig. 3). All fractures achieved union without loss of reduction in both groups. No cast breakage occurred in either group. However, there were more skin complications in the fiberglass casting group (17) than in the biobased polyester group (5), which was statistically significant (p = 0.015) (Table 3). All skin complications resolved uneventfully after cast removal.

**Table 2:** Patient demographics

Variable	Biobased polyester (N = 50)	Fiberglass (N = 50)	p-value
Age (years)	7.62	7.89	0.6904
Sex			0.9454
Male	34	35	
Female	16	15	
Fracture site (N) Radius/ulna Distal humerus	27 23	26 24	0.9494
Duration of casting (days)	24.9 ± 5.90	25.2 ± 6.72	0.6874

**Table 3:** Complications in the biobased polyester group and fiberglass group

Variable	Biobased polyester	Fiberglass	p-value
Nonunion	0	0	
Loss of reduction	0	0	
Cast breakage	0	0	
Skin problem	5	17	0.015
Total complications	5	17	0.015

## Discussion

The single-center randomized controlled study, which included both patient reported satisfaction rates and clinical parameters, showed that the novel biobased polyester cast provided similar clinical outcomes but better patient satisfaction scores than the synthetic fiberglass cast for the treatment of stable upper limb fractures in children.

The traditional synthetic cast contains toxic components, such as isocyanates, and prolonged skin contact may result in itching, redness, and dryness. Animal studies have shown that skin exposure to chemical allergens can also induce asthmatic responses [10]. Occupational asthma after exposure to casts containing methylene diphenyl diisocyanate has been reported [11]. Work-related asthma was found in two people in Finland due to long-term exposure to fiberglass casts and subsequent cyanate-vaporization byproducts [12]. Some casts can be classified as hazardous waste which increases the environmental burden.

**Fig. 4:**
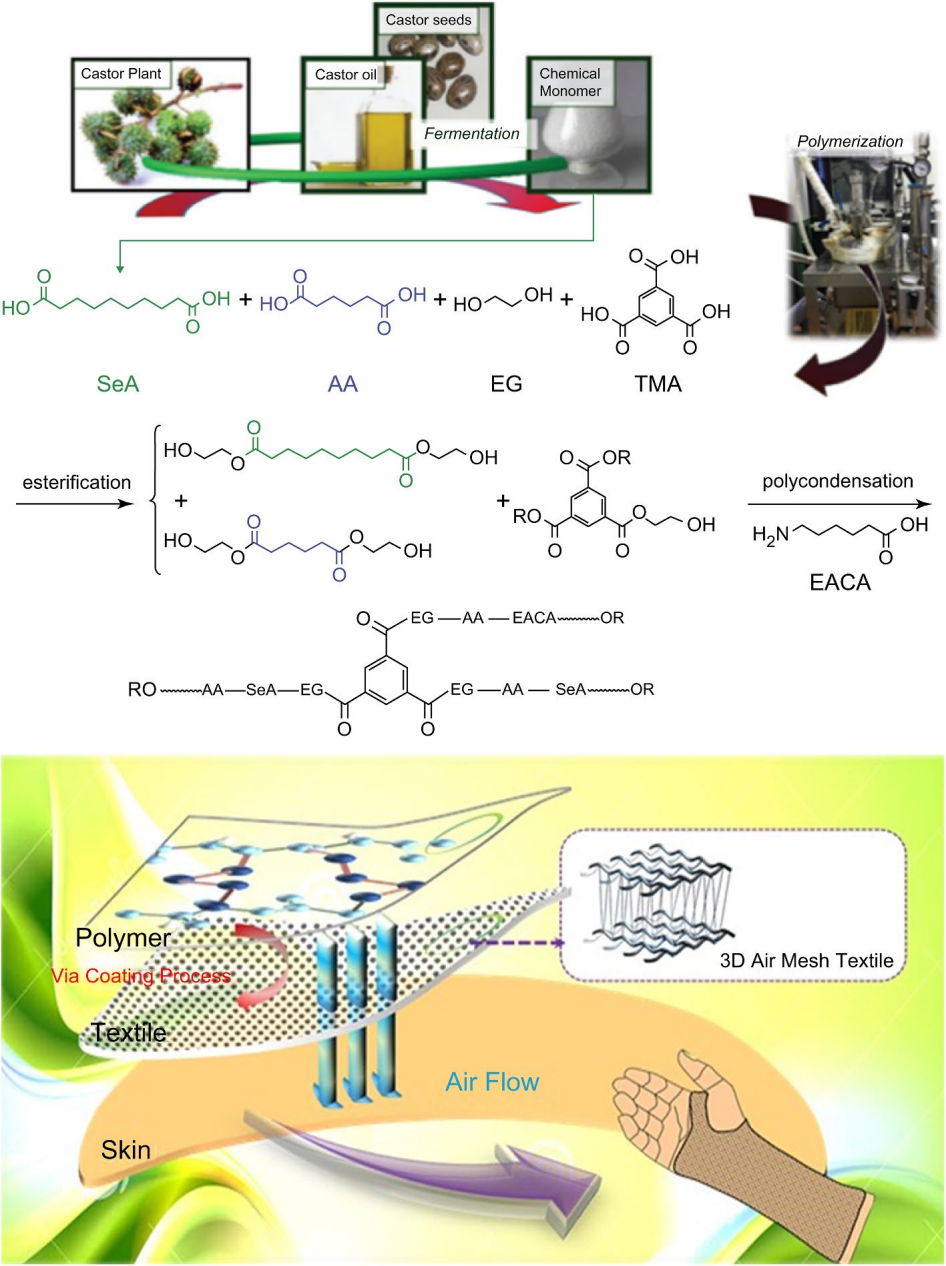
(**A**) Synthesis of the poly(ethylene sebacate-co-ethylene adipate). copolyester. Abbreviations: SeA = sebacic acid, EG = ethylene glycol, AA = adipic acid, TMA = trimesic acid, and EACA = aminocaproic acid. (**B**) The schematic picture displays the composite of the novel biobased polyester cast

The novel biobased material used in this study was made using a newly developed nontoxic and biodegradable copolymer called poly(ethylene sebacate-co-ethylene adipate) (PESA) synthesized from sebacic acid, a green resource, that is copolymerized with ethylene glycol, trimesic acid, aminocaproic acid, and adipic acid (Fig. 4A) [8]. A 3D air mesh fabric was coated with the PESA co-polyester, forming a composite material which could be shaped easily after soaking in 60 °C or by heating for 20 s with a hairdryer. After cooling to room temperature, the polymer chain of the PESA copolyester could easily be packed into an ordered state to maintain the supporting given by the 3D mesh fabric in order to form a rigid composite material (Fig. 4B).

Due to the notoriously poor compliance capabilities of children, long arm casting is still the superior choice for immobilization than removable braces and splints for treating stable upper limb fractures. Synthetic fiberglass casts were preferred over POP, as they are more water-resistant, lighter, and tougher [13]. However, skin irritation and itchiness remain a major complaint that can lead to patient dissatisfaction, especially in humid regions. The novel design of the biobased polyester cast includes larger ventilation holes to allow for additional airflow to the skin, which decrease the frequency of skin irritation and itchiness. A study indicated that using a waterproof cast to encourage activities resulted in a 10% higher performance on the Activities Scale for Kids compared to traditional fiberglass casts [14]. Both materials in our study were water resistant; however, in clinical practice, we did not allow patients to wet the cast due to the humid climate and unpredictable patient compliance. In this study, both groups were not allowed to wet the cast and non-waterproof padding material was used before casting to eliminate potential bias and confounding of the results. Itchiness or skin irritation is a significant contributor to overall patient satisfaction. The biobased polyester cast had large ventilation holes which allowed for better airflow and may contribute to less itching, less odor, and better overall patient satisfaction.

**Fig. 5:**
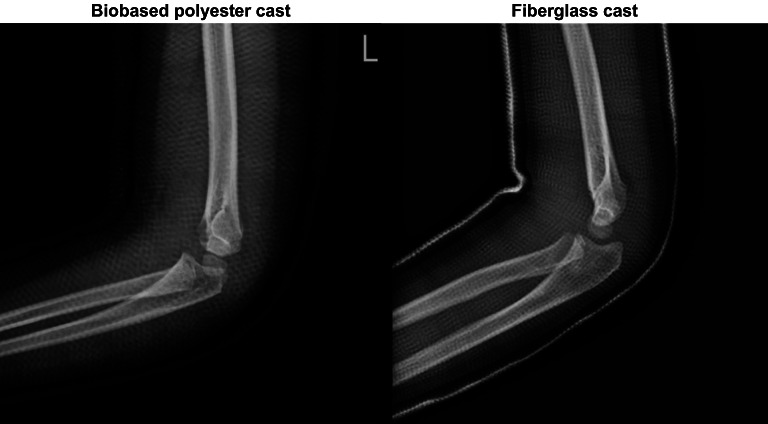
A radiography image comparing transparency between the two groups. (**A**) Biobased polyester cast; (**B**) Fiberglass cast

The biobased cast was light and translucent like fiberglass, which allowed for visualization of the bone on regular plain film (Fig. 5). However, there were shortcomings when using the novel biobased cast. First, it should be applied and molded with hot water over 3–5 min to become malleable, which requires more equipment needing to be prepared in the plaster room. The fiberglass cast was easier and more convenient to apply than the novel one. Second, the edge of the biobased cast was sharper than the fiberglass cast, which needed to be covered with reversal cotton stockinet on the surface. Despite the shortcomings of the novel cast composition, the overall patient satisfaction was significantly superior to the fiberglass cast.

This study has some limitations. First, we relied on a single questionnaire to assess patient satisfaction, which could introduce issues related to the reliability and validity of our evaluation. However, few scoring systems are available that fit our needs. Second, we included the most common upper limb fractures in children, while other studies only included distal radius fracture [4, 15]. Although the distribution of fractures between the groups was similar, there is the potential for a sampling error. Third, fiberglass casting material can be done with synthetic padding, which has breathability and causes less skin irritation in a moist environment. It is waterproof, to allow for swimming and bathing, and mitigates the difficulties of skin rashes in children who did not comply with keeping a cast dry. Using cotton padding and stockinette with fiberglass casting takes away many of the advantages of the fiberglass. Fourth, we did not perform the cost analysis in this study. This new material is a novel composition, and unlike the traditional synthetic cast, the price was floating. We found it difficult to assign a price based on increased comfort and a positive treatment experience.

## Conclusions

In conclusion, the novel biobased cast is nontoxic and recyclable. It provided similar clinical outcomes to traditional fiberglass casts, reduced itchiness, and provided better overall patient satisfaction during treatment of stable upper limb fractures in children.

## Data Availability

The datasets used and/or analysed during the current study available from the corresponding author on reasonable request.

## Abbreviations

POP: plaster of Paris
PESA: poly(ethylene sebacate-co-ethylene adipate)
